# Anti-Bacterial and Anti-Inflammatory Effects of Toothpaste with Swiss Medicinal Herbs towards Patients Suffering from Gingivitis and Initial Stage of Periodontitis: From Clinical Efficacy to Mechanisms

**DOI:** 10.3390/dj8010010

**Published:** 2020-01-15

**Authors:** Zaira F. Kharaeva, Magomet Sh. Mustafaev, Anzor V. Khazhmetov, Ismail H. Gazaev, Larisa Z. Blieva, Lukas Steiner, Wolfgang Mayer, Chiara De Luca, Liudmila G. Korkina

**Affiliations:** 1 Department of Microbiology, Virology and Immunology, Kabardino-Balkar Berbekov’s State University, 176 Chernishevskogo St., 360000 Nal’chik, Russia; irafe@yandex.ru (Z.F.K.); mouette@yandex.ru (L.Z.B.); 2 Department of Dentistry & Maxillofacial Surgery, Kabardino-Balkar Berbekov’s State University, 176 Chernishevskogo St., 360000 Nal’chik, Russia; musmag@mail.ru (M.S.M.); andzorhazhmetov@mail.ru (A.V.K.); 3 Department of Molecular Diagnostics, Russian Federation State Reference Centre for Phyto- and Veterinary Control, 1 Ninth May St., 360000 Nal’chik, Russia; is.gazaev@yandex.ru; 4 Marketing Department, TRISA AG, 31 Kantonstrasse, CH-6234 Triengen, Switzerland; Lukas.Steiner@trisa.ch; 5 R&D Department, MEDENA AG, 16 Industriestrasse, 8910 Affoltern-am-Albis, Switzerland; wolfgang.mayer@medena.ch (W.M.); chiara.deluca@medena.ch (C.D.L.); 6 Centre of Innovative Biotechnological Investigations Nanolab (CIBI-NANOLAB), 197 Vernadskiy Pr., 119571 Moscow, Russia

**Keywords:** anti-bacterial effects, anti-inflammatory effects, bacterial catalase, gingival crevicular fluid, gingivitis, herbal extracts, interleukins, nitric oxide, periodontitis, total antioxidant activity

## Abstract

Objective: To distinguish clinical effects and mechanisms of sodium monofluorophosphate plus xylitol and herbal extracts of Swiss medicinal plants (*Chamomilla recutita*, *Arnica montana*, *Echinacea purpurea*, and *Salvia officinalis*). Materials and Methods: A 2-month-long comparative clinical study of toothpaste containing 1450 ppm sodium monofluorophosphate and xylitol (control, 15 patients) and toothpaste additionally containing extracts of the medicinal herbs (experiment, 35 patients) was performed on patients with gingivitis and the initial stage of periodontitis. Clinical indices of gingivitis/periodontitis were quantified by Loe & Silness’s, CPITN, OHI-S, and PMA indexes. The pro-inflammatory and anti-inflammatory interleukins, nitrites/nitrates, total antioxidant activity, and bacterial pattern characteristic for gingivitis and periodontitis were quantified in the gingival crevicular fluid and plaque. In the in vitro tests, direct anti-bacterial effects, inhibition of catalase induction in *Staphylococcus aureus*, in response to oxidative burst of phagocytes, and intracellular bacterial killing were determined for the toothpastes, individual plant extracts, and their mixture. Results: Experimental toothpaste was more efficient clinically and in the diminishing of bacterial load specific for gingivitis/periodontitis. Although the control toothpaste exerted a direct moderate anti-bacterial effect, herbal extracts provided anti-inflammatory, anti-oxidant, direct, and indirect anti-bacterial actions through inhibition of bacterial defence against phagocytes. Conclusions: Chemical and plant-derived anti-bacterials to treat gingivitis and periodontitis at the initial stage should be used in combination amid their different mechanisms of action. Plant-derived actives for oral care could substitute toxic chemicals due to multiple modes of positive effects.

## 1. Introduction

The regular use of adequate toothpastes with safe active ingredients possessing anti-bacterial, anti-inflammatory, anti-oxidant, and regenerative properties is one of the most effective strategies for prevention and treatment of gingivitis, periodontal pathologies, and caries leading to teeth loss.

The mouth, like other areas of the digestive tract, possesses a natural microflora, the presence of which confers several beneficial properties to the host. However, in the absence of adequate oral hygiene, dental plaque can accumulate beyond levels compatible with oral health. This leads to shifts in the balance of the predominant bacteria away from those associated with health; such shifts can predispose a site to caries, gingivitis, or periodontal diseases [1]. Possible strategies to maintain the stability and beneficial properties of the natural oral microflora include improvements to oral hygiene, for example, by using products containing safe anti-plaque antimicrobial anti-inflammatory and anti-oxidant substances.

Periodontitis is a chronic inflammatory infectious pathology caused by dental plaque bacteria. The infection-induced inflammatory process leads to progressive destruction of the tissues supporting the teeth, such as the gum, periodontal ligament, cementum, and alveolar bone. Periodontitis is currently regarded as a dysbiotic inflammatory disease with a negative impact on both oral and extra-oral sites [2,3]. Gingivitis is a common non-destructive form of periodontal disease, since periodontitis is always an outcome of precedent gingivitis, although gingivitis under circumstances could resume without evolution to periodontitis [4]. High throughput methods, such as proteomics and genomics, facilitated discriminative and quantitative analyses of oral microbiota. For example, prevalence of *Fusobacterium nucleatum* and *Prevotella oulorum* species has been associated with gingivitis [5].

The chronic inflammation of periodontal tissues is induced by poly-microbial “complexes” [6,7,8], i.e., associations forming biofilms in gingival pockets and supra-gingival plaque [9]. In such biofilms, microbes are more pathogenic and less sensitive to traditional antibiotic therapies [10] owing to their innate or/and acquired capacity of adaptation to both anti-microbial drugs and host defence [11,12,13,14].

A few of the numerous microbes residing in the oral cavity, mainly anaerobic Gram-negative and Gram-positive bacteria, may cause periodontal pathology. For example, a group of periodontal pathogens of high risk consists of *Aggregatibacter actinomycetemcomitans (A.a.)*, *Porphyromonas gingivalis (P.g.)*, *Tannerella forsythensis (T.f.)*, and *Treponema denticola* (*T.d.*), while pathogens of moderate risk in concentrations higher than the threshold are *Porphyromonas endodontalis (P.e.)*, *Fusobacterium nucleatum (F.n.)*, and *Prevotella intermedia (P.i.)* [15,16,17].

The presence of distinct microbes in the periodontal environment has been associated with increased levels of host-produced pro-inflammatory cytokines, such as tumour necrosis factor α (TNF-α), interleukin 6 (IL-6), and interleukin 17A (IL17A) in the gingival fluid and tissue, that define the severity of destructive processes in the gingival epithelium and bone tissue [7]. Interleukin-1beta, a potent stimulator of bone resorption, was hypothesised to be implicated in the pathogenesis of periodontal tissue destruction [18], while IL-17 is implicated in the evolution of gingivitis into periodontitis [6]. Therefore, any safe mean suppressive to the oral pattern of pro-inflammatory and anti-inflammatory interleukins could be efficient in the treatment of gingivitis and could also prevent a transition from gingivitis to periodontitis. It has become common knowledge that infection-induced chronic inflammation is closely associated with an imbalance of reactive oxygen/nitrogen species and antioxidant defence; so called oxidative stress [19,20]. For example, chronically elevated levels of gingival pro-inflammatory cytokines (gingivitis and periodontitis) [21] are always associated with severe local and generalised oxidative stress [22,23,24,25,26].

Anti-bacterial chemical oral care products with chlorhexidine, fluorides [27,28], xylitol [29,30], triclosan [28], and their combinations [31] have demonstrated a decrease of the bacterial count in the in vitro systems, including bacterial biofilms, as well as in vivo anti-bacterial, anti-caries, and anti-inflammatory effects. However, concerns regarding their absorption, retention, and multi organ toxicity have been steadily growing. Fluoride-containing substances that are practically ubiquitous in modern toothpastes and mouth washes have a low safety profile, especially in children and pregnant women [32]. Molecular mechanisms of fluoride toxicity have been reviewed in a recent publication [33].

The most spectacular example of chemical antiseptics is triclosan, which is still widely utilised in oral care products, with the claim to fight gingivitis [34,35,36].

A great public and medical concern has been raised due to the use of triclosan—containing products, since the plasma levels of it increased rapidly [37], followed by disruption of essential biological processes [38] through numerous molecular pathways (Reviewed in [38,39,40]).

At the same time, recent studies have shown periodontitis and plaque/gingivitis-controlling effects of fully herbal toothpastes and gingival gels [41] comparable to the effects of chemical anti-microbial toothpastes [42]. Several medicinal herbs included into Swiss Pharmacopoeia have acceptable safety profiles and remarkable health effects that are being applied topically. Among them are flower heads of *Arnica montana*, included in many Pharmacopoeias [43,44], with detailed phytochemical analysis [44,45,46]. Chamomile (*Chamomilla recutita*) is one of the 12 most used medicinal herbs known for its therapeutic properties [47] and is included in all European Pharmacopoeia. The extracts of the flowers are added to numerous topical compositions due to their established anti-inflammatory and anti-oxidant effects [48]. Antimicrobial action of *Chamomilla recutita* is mainly ascribed to sesquiterpene alpha-bisabolol [47].

In the recent review [49], pharmacological properties of *Salvia officinalis* and their correlation with phytochemical content have been discussed in detail. The German Commission E has accepted medicinal applications of *Salvia officinalis* exclusively to suppress inflammation in oral cavity and skin [50]. The aerial parts of *Salvia officinalis* contain a plethora of secondary metabolites with anti-inflammatory, anti-oxidant, anti-microbial properties [49,51,52,53].

*Echinacea purpurea* has been traditionally used in folk medicine to treat infections and accelerate wound healing. Its extract exerted more relevant and long-lasting anti-microbial effects than chlorhexidine [54]. Active constituents of *Echinacea* flowers induced macrophages to enhance phagocytosis and intracellular bacterial killing [55] and possessed remarkable antioxidant [56] and anti-inflammatory [57] properties. 

In the present clinical laboratory study, we evaluated the clinical efficacy of the toothpaste containing chemical anti-bacterial substances (sodium monofluorophosphate (1450 ppm) and Xylitol) and four medicinal plant extracts (*Arnica montana*, *Salvia officinalis*, *Chamomilla recutita*, and *Echinacea purpurea*) in a group of patients with gingivitis and initial stages of periodontitis. As a control, toothpaste without herbal extracts was used. The laboratory part of the clinical study was designed to distinguish anti-bacterial effects of chemical and plant-derived constituents of the toothpaste and to elucidate mechanisms, by which these anti-bacterial effects were achieved. Comparative evaluation of redox balancing and anti-inflammatory effects of the experimental and control toothpastes was also carried out.

## 2. Materials and Methods

### 2.1. Products for Examination

The toothpaste under examination (experimental toothpaste (ETP), Trisa Revital Sensitive, manufacturer TRISA AG, Triengen, Switzerland) contained the following active ingredients: sodium monofluorophosphate (1450 ppm), xylitol, and Swiss medicinal herbs. Herbal active ingredients were aqueous-ethanol extracts of the medicinal herbs from Swiss Pharmacopoeia, such as *Chamomilla recutita* leaves (containing no less than 0.1% of alpha-bisabolol), *Salvia officinalis* leaves (containing no less than 10% of total phenols), *Arnica montana* flowers (containing no less than 0.04% sesquiterpene lacton), and *Echinacea purpurea* flowers (containing no less than 1% echinacoside). The control toothpaste (CTP) contained the same excipients as the ETP plus sodium monofluorophosphate (1450 ppm) and xylitol as actives. These two toothpastes were used for clinical and laboratory evaluation in order to distinguish clinical and biological effects of herbal constituents from those of fluoride and xylitol.

In the in vitro experiments, individual extracts of *Chamomilla recutita* leaves, *Salvia officinalis* leaves, *Arnica montana* flowers, and *Echinacea purpurea* flowers (all purchased from Biologica AG, Switzerland), or their mixture in proportions used in the toothpaste, were added to bacteria to show their direct anti-bacterial effects. They were also added to bacteria before phagocytosis by human granulocytes to evaluate their effects towards bacterial catalase and intracellular bacterial killing.

### 2.2. Patients and Study Design

The study enrolled a group of 50 patients of both sexes (age range 35–55 years) suffering from gingivitis or initial stages of periodontitis and visiting dentists at Dentistry and Maxillofacial Surgery Department of the Kabardino-Balkar Berbekov’s State University (Nal’chik, Russian). The study protocol was scrutinized and approved by the local Ethical Committee (Protocol MD-023-2017). The patients were randomly assigned to experimental or control groups. The demographic distribution of periodontitis patients in the groups is shown in Table 1.

All of them were treated by traditional hygienic and therapeutic protocols, if needed. Traditional treatment protocols included education to oral hygiene, plaque removal, teeth enamel polishing, and elimination of tartar, if needed. All recruited patients agreed to not use any toothpaste for 72 h prior to the clinical study (wash-out period).

The patients of the control (n = 15) and experimental (n = 35) groups were recommended to brush teeth with CTP or ETP, respectively, two times a day for 60 consecutive days. The samples of toothpastes were distributed to the participants free of charge. The patients were instructed how to brush teeth. The tubes with toothpastes were numerated and patients were not informed whether they were using placebo or experimental toothpaste. Laboratory staff and medical doctors who were carrying out the measurements and clinical assessment procedures were not informed about the use of either CTP or ETP. On these grounds, this pilot clinical study was characterised as a double-blind placebo-controlled study.

Healthy donors matched by sex and age (n = 25) were recruited from the Medical Department staff and trainees, who donated gingival crevicular fluid (GCF). The normal ranges of different markers in GCF derived from the measurements performed on this biological material.

Subjects with severe chronic and/or infection diseases in the acute phase, as well as virus hepatitis patients, were excluded from the study. No patients or controls entering the study had taken any drugs or nutraceutical supplements known to interfere with the redox status or inflammation for at least six weeks. No alcohol- or drug-abusers were present in any of the three cohorts studied. Five smokers were in the experimental group and three smokers were in the control group. All subjects consented to personal and anamnestic data collection and biological material sampling.

### 2.3. Clinical Assessment

Clinical efficacy of ETP and CTP was assessed by subjective evaluation of doctors and patients and objective clinical indices of gingivitis and chronic mild periodontitis [58,59]. These indices included the gingival and plaques indexes, in accord with Löe & Silness’s method, the state of gingival inflammation by Parma’s papillae-gum margin-alveolar (PMA) index, the International CPITN test, and the OHI-S index. All the indexes were determined twice at days 0 and 60 of the clinical study.

The Periodontal World Health Organisation index defines the need of therapy against periodontal pathologies of any type. With the help of a special graduated periodontal probe, the clinical state of gingival sulcus and periodontal tissue in the vicinity of six teeth was registered and expressed as a score: 0—absence of pathology; 1—bleeding after the probe introduction; top of the gum is slightly inflamed; 2—pathological gingival pocket of 4–5 mm in depth; 3—pathological gingival pocket of 6 mm and more in depth. The final result was calculated from the ratio of the score sum divided by 6. The clinical significance of the CPITN score was as follows: 0—no therapy needed; 1—instructions on individual oral hygiene are needed; 2–3—professional oral/teeth hygiene is needed plus instruction on individual oral hygiene; 4—complex therapy of periodontal tissues is necessary.

The PMA index allows semi-quantitative assessment of the gum state and diagnosis of gingivitis. Gums were stained by a special non-toxic dye and the PMA score was determined by analysis of the dye penetration into gingiva: 0—no penetration, no inflammation; 1—moderate inflammation of gingival papilla (P); 2—inflammation of marginal gum (M); 3—inflammation of alveolar gum (A). The PMA index was expressed in % and calculated by the formula:

PMA = ∑ scores/3 × number of teeth × 100%.

The clinical significance of PMA defines: 30%—mild gingivitis; 31–60%—medium gingivitis; 61% and more—severe gingivitis.

Index OHI-S is a simplified Green-Vermillion index introduced in 1964. The index quantifies the state of oral hygiene by the measurement of teeth surface covered by plaque or/and tartar: OHI-S = ∑ (TP/n) + ∑(TT/n), where n is a number of teeth: teeth plaque (TP); teeth tartar (TT).

The Silness–Loe Index assesses the thickness of the plaque close to the gum by a score from 0 to 3.

### 2.4. Biological Material Collection and Processing

A Whatman no. 1 sterile paper bar of 3 mm width was carefully introduced into the tooth pocket, or into the gingival sulcus and kept in place for 2 min. The filter paper bar was then transferred into vials containing 2 mL phosphate buffer solution. The procedure was carried out for several affected teeth, and the content of vials was finally pooled. The collected samples of gingival crevicular fluid (GCF) were stored at −80 °C until they were analysed for differential microbial counts by real-time PCR, for nitrite/nitrate and cytokine contents, and for total antioxidant activity determination.

Plaque was collected from six teeth by a sterile dental probe and mixed with GCF of the same patient to be further examined by qrPCR.

Peripheral venous blood (20 mL) was drawn after overnight fasting into vacutainers with ethylene diamine tetra-acetic disodium salt (EDTA) as the anti-coagulant. Both patient and donor samples were processed and analysed in parallel. Circulating polymorphonuclear leukocytes (PNM) were obtained by double density gradient centrifugation of 15 mL of total blood (Histopaque, *d* = 1.077 and 1.199 g/mL). PMN from the interface were re-suspended in phosphate buffer saline, centrifuged at 1650 rpm for 10 min, and then aliquoted at 5 × 10^6^/vial. Freshly isolated PMN were used in phagocytosis assays, intracellular microbial killing tests, and microbial catalase activity determination [41].

### 2.5. Reagents and Assay Kits

The majority of chemical reagents and solvents, H_2_O_2_ standard, and mediums for human and bacterial cell cultivation were purchased from Sigma Chemical Co. (St. Louis, MO, USA); kits for enzyme activities and nitrite/nitrate assays were from Cayman Chem. Co. (Ann Arbor, MI, USA); monoclonal antibodies for enzyme-linked immunosorbent assay (ELISA) interleukin kits were from R&D Systems (Minneapolis, MN, USA); kits for protein determination were from Bio-Rad Laboratories (Bio-Rad Inc., Hercules, CA, USA).

### 2.6. Bacterial Strains and Growth Conditions

Bacterial strains used in the in vitro study were 10 different strains of *Staphylococcus aureus* isolated from oral and nasal cavities. The strains are collected in Table 1. *S. aureus* was grown in tryptic soy broth at 37 °C under continuous shaking, as described previously [60].

### 2.7. Phagocytosis and Post-Phagocytosis Bacterial Survival Assays

Intensity of phagocytosis was assessed by routine clinical bacteriology methods. Briefly, 1 mL of PMN suspension (10^6^ cells/mL) was mixed with 1 mL of bacterial suspension (10^7^ cells/mL). The mixture was incubated under continuous shaking at 37 °C for 30 min. Smears were then prepared on microscopic slides, fixed, and stained by Romanovsky–Giemsa dye. The smears were examined under a microscope, and the % of phagocyting PMN was determined. The remaining mixture was used to assess intracellular bacterial killing. After centrifugation at 1500× g for 10 min, bacterial sediments were collected and diluted to an OD_600_ of 0.1 with fresh medium, spread onto Petri dishes with appropriate agar-containing medium, and were allowed to grow at 37 °C for 24 h. Bacteria survival rates were calculated as colony-forming-units (CFU) of cells, co-incubated with granulocytes and divided for that of untreated bacteria. The results were expressed in % [41].

In the in vitro experiments with toothpastes and active ingredients, the following procedure was applied. One mL of bacterial cells (*S. aureus*, strain 1823) containing 1.5 × 10^9^ cells/mL was mixed with 100 μL of 0.9% toothpaste solution in a physiological medium or 10 μL of the individual plant extract or 10 μL of the extract mixture prepared in accord with their composition in ETP. In the control cultures, 100 or 10 μL physiological solution was added, respectively. The cultures were incubated at 37 °C for 60 min. Bacterial suspensions were then thoroughly washed and used for the analyses of phagocyte activity and intracellular killing.

### 2.8. Bacterial Catalase Assay

Bacterial cells pre-treated and non-pretreated with ETP or CTP, or active herbal ingredients of ETP, were grown in appropriate mediums to an OD_600_ of 0.3 (photo in Table 1, sectors indicate MN1—CTP; MN2—ETP; A, C, E + P, and S—*Arnica montana*, *Chamomile recutita*, *Echinacea purpurea*, and *Salvia officinalis*, respectively). The bacterial layer was scraped from the bottom of the Petri dish, collected together with the culture medium, centrifuged, and lysed with a Pierce bacterial extraction reagent. Cell debris was removed by centrifugation, and catalase activity in the supernatant was measured by Aebi’s method [61], using freshly prepared H_2_O_2_ for a standard curve. The protein content was measured according to Bradford’s method, using the Bio-Rad micro-plate assay Kit (Bio-Rad Laboratories, Inc. (Hercules, CA, USA)). One unit of catalase activity was defined as the decomposition of 1 μmol H_2_O_2_ per min at pH 7.0 at 25 °C. The results were expressed in Units/g protein.

In the in vitro experiments with toothpastes and active ingredients, the following procedure was applied. One mL of bacterial cells (*S. aureus*, strain 1823) containing 1.5 × 10^9^ cells/mL was mixed with 100 μL of 0.9% toothpaste suspension in the physiological medium, 10 μL of the individual plant extract, or 10 μL of the plant extract mixture, prepared in accord with their composition in ETP. In the control cultures, 10 or 100 μL physiological solution was added. The cultures were incubated at 37 °C for 30 min. Bacterial suspension was then thoroughly washed and used for the catalase test.

### 2.9. Differential Bacterial Concentrations in GCF and Plaque Determined by a Quantitative Real-Time Reverse Transcription Polymerase Chain Reaction (qrPCR) Method

DNA was isolated from samples of GCF and plaque and kept on ice for no more than 12 h. DNA was amplified with iQ^TM^ Supermix using the MiniOpticon Real-Time PCR Detection System (Bio-Rad, Hercules, CA, USA). All real-time assays were carried out under the following conditions: 35 cycles of denaturation at 95 °C for 15 s; annealing and extension at 60 °C for 60 s. Melt curve analysis was performed to confirm the specificity of the amplified products. All samples were run in triplicate, and relative expression was determined by normalizing samples to housekeeping genes. The primers corresponding to seven periodontal pathogens of high and medium risk were used (*Aggregatibacter actinomycetemcomitans*, *Porphyromonas gingivalis*, *Tannerella forsythensis*, *Treponema denticola, Porphyromonas endodontalis*, *Fusobacterium nucleatum*, and *Prevotella intermedia*). Results were expressed as the absolute count of definite bacterial cells in 1 mL of GCF. The qrPCR analysis was performed two times (in the beginning and at the succession of the clinical study) on 15 patients of the experimental group and 5 patients of the placebo group.

### 2.10. Reduction-Oxidation (Redox) Assays

The GCF levels of nitrites/nitrates (NO_2_^−^/NO_3_^−^, expressed as μmoles/L or μM) were measured spectrophotometrically by Griess reagent Kit, following the manufacturer’s instructions. The total antioxidant activity (total AOA) of GCF was measured by the method described elsewhere. In brief, 100 μL of egg yolk was mixed with 10 μL GCF, collected from gingival sulcus or a periodontal pocket. Then, 100 μL of FeSO_4_ was added and the volume was adjusted to 1 mL by a physiological solution. The mixture was incubated at room temperature for 30 min, and 0.5 mL of 20% trichloroacetic acid plus 0.1 mL of 0.01 M butyl hydroxy toluol (ionol) in ethyl alcohol were added. The tubes were centrifuged at 1500× *g* for 10 min and supernatant was collected. The mixture of 0.7 mL of supernatant and 0.6 mL of 0.5% thiobarbituric acid (TBA) was heated at 100 °C for 30 min, cooled down, and an absorbance at a wave length of 532 nm was determined. Antioxidant activity was expressed in % of the control samples without biological material.

### 2.11. Cytokine Assays

The GCF levels of pro-inflammatory interleukins 1beta (IL-1β), IL-6, IL-17, and anti-inflammatory interleukin 10 (IL-10) were measured by enzyme-linked immunosorbent assay (ELISA) purchased from R&D Systems (Minneapolis, MN, USA), following the manufacturer’s instructions. Cytokine concentrations were expressed in pg/mL of GCF, and each protein factor was quantified in the linear range of its calibration curve.

### 2.12. Statistical Analysis

All biochemical measurements were done in triplicate, and data were statistically evaluated. Statistical analysis of clinical and laboratory data was performed using the STATISTICA 6.0 program (StatSoft Inc., Tulsa, OK, USA). Reported data were treated as continuous. Normality of data was checked using the Shapiro–Wilk test. Since the distribution of the data in the groups was significantly different from normal, non-parametric statistics was used. Values were presented as mean ± standard error of the mean of triplicate analyses. The Mann–Whitney U-test for independent samples was employed for comparison between placebo and experimental groups. Significance was assumed at a *p*-value of <0.05. This section may be divided by subheadings. It should provide a concise and precise description of the experimental results, their interpretation and the experimental conclusions that can be drawn.

## 3. Results

### 3.1. Clinical Efficacy of Experimental and Placebo Toothpastes

All patients of the experimental group noticed a pleasant taste and fragrance of the ETP, the slight but pleasant foaming of the toothpaste, the diminished bleeding of gums (32 patients), and teeth whitening (17 patients). Patients of the control group also observed a pleasant taste and slight foaming of CTP, although none of them reported any clinical effect.

**Figure 1:**
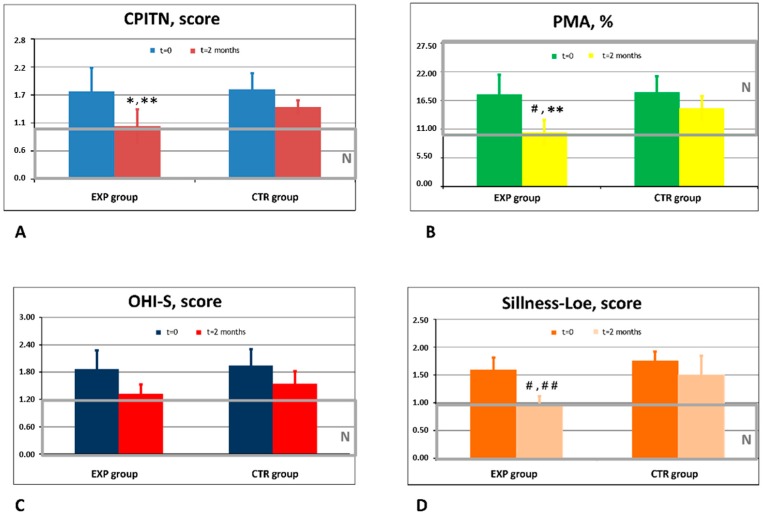
Effects of experimental (ETP) and control toothpaste (CTP) on clinical markers of gingivitis and periodontitis. (**A**) Dynamics of the CPITN score in the experimental (EXP) and control (CTR) groups of patients. *N*-normal range of values obtained in healthy people. * *p* < 0.05 vs. baseline values; ** *p* < 0.05 vs. CTR. (**B**) Dynamics of the PMA index in the experimental (EXP) and control (CTR) groups of patients. *N*-normal range of values obtained in healthy people. ^#^ *p* < 0.01 vs. baseline values; ** *p* < 0.05 vs. CTR. (**C**) Dynamics of t hOHI-S score in the experimental (EXP) and control (CTR) groups of patients. N-normal range of values obtained in healthy people. (**D**) Dynamics of the gingival and plaque Sillness–Loe score in the experimental (EXP) and control (CTR) groups of patients. N-normal range of values obtained in healthy people. ^#^ *p* < 0.01 vs. baseline values; ^##^ *p* < 0.01 vs. CTR.

Doctor-investigators noticed high clinical efficacy of ETP, and their observations fully corresponded to the data of objective instrumental investigations (Figure 1).

Recruited patients assigned to both control and experimental groups exhibited clinical features of gingivitis and the initial stage of periodontitis that was revealed by the assessment with conventional objective indexes of gingival and periodontal conditions (Figure 1).

The baseline values of the four clinical indexes did not differ in the experimental and control groups. The indexes of the gingivitis, general oral hygiene, plaque, and tartar presence and mild periodontitis subsided during a 60 day use of both ETP and CTP. The effects assessed objectively by four indexes: (CPITN, PMA, OHI-S, and Loe & Sillness) were statistically significantly greater in the EPT group as compared to the CTP group (*p* < 0.05). These indexes reached normal levels in the EPT group by the end of the study, while indexes in the CTP group still exceeded normal values (Figure 1A,B,D).

### 3.2. Effects of ETP and CTP on Pro- and Anti-Inflammatory Cytokines in GCF

**Figure 2:**
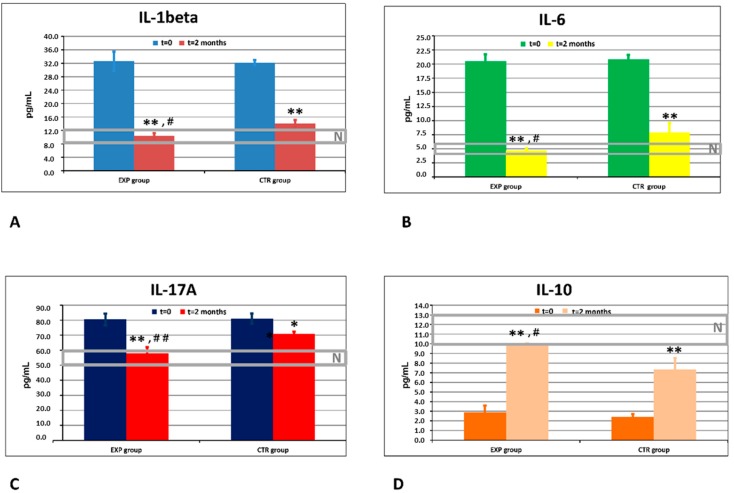
Effects of ETP and CTP on inflammatory cytokines in gingival crevicular fluid (GCF). (**A**) Dynamics of cytokine IL-1beta (pg/mL) in the experimental (EXP) and control (CTR) groups of patients. *N*: normal range of values obtained in healthy people. ** *p* < 0.01 vs. baseline values; ^#^ *p* < 0.05 vs. CTR. (**B**) Dynamics of cytokine IL-6 (pg/mL) in the experimental (EXP) and control (CTR) groups of patients. *N*: normal range of values obtained in healthy people. ** *p* < 0.01 vs. baseline values; ^#^ *p* < 0.05 vs. CTR. (**C**) Dynamics of cytokine IL-17A (pg/mL) in the experimental (EXP) and control (CTR) groups of patients. *N*: normal range of values obtained in healthy people. * *p* < 0.05 vs. baseline values; ** *p* < 0.01 vs. background values; ^##^ *p* < 0.01 vs. CTR. (**D**) Dynamics of cytokine IL-10 (pg/mL) in the experimental (EXP) and control (CTR) groups of patients. *N*: normal range of values obtained in healthy people. ** *p* < 0.01 vs. baseline values; ^#^ *p* < 0.05 vs. CTR.

Quantification and dynamics of GCF levels of three pro-inflammatory cytokines (IL-1β, IL-6, and IL17A), and of the anti-inflammatory cytokine IL-10, are shown in Figure 2A–D, respectively, and compared with the corresponding cytokine levels in healthy subjects. In the GCF, the background levels of all pro-inflammatory cytokines, IL-1β, IL-6, and IL17A, were highly and equally elevated, while IL-10 values were decreased in both experimental and control groups as compared to healthy controls. In the course of the clinical study, the GCF cytokine concentrations reached normal levels by day 60 in the patients of the ETP group, while normal GCF cytokine levels were never achieved in the control group. Differences between the groups were statistically significant (*p* < 0.05).

### 3.3. Comparison of In Vivo Effects of ETP and CTP on Nitrite/Nitrate Content and Total Antioxidant Activity in GCF

**Figure 3:**
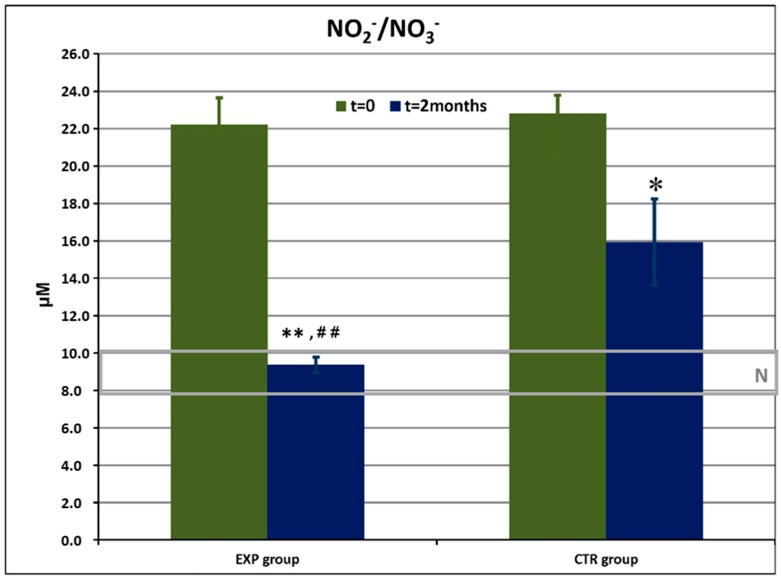
Nitrite and nitrate (NO_2_^−^/NO_3_^−^) levels (μM) in gingival crevicular fluid in the experimental (EXP) and control (CTR) groups of patients before and after the trial. N: normal range of values obtained in healthy people. * *p* < 0.05 vs. baseline values; ** *p* < 0.01 vs. baseline values; ^##^ *p* < 0.01 vs. CTR.

The dynamics of nitrite/nitrate levels in GCF is shown in Figure 3.

The data of recruited patients were compared with those of healthy donors (n = 25) matching in age and sex. The levels of NO_2_^−^/NO_3_^−^ in GCF were highly elevated in both experimental and control groups vs normal values. Baseline concentrations of NO_2_^−^/NO_3_^−^ did not differ for the experimental and control groups. The levels of this pro-inflammatory marker were remarkably diminished by the 60th day in the ETP group, reaching normality range of values, and remained higher-than-normal in the control group.

**Figure 4:**
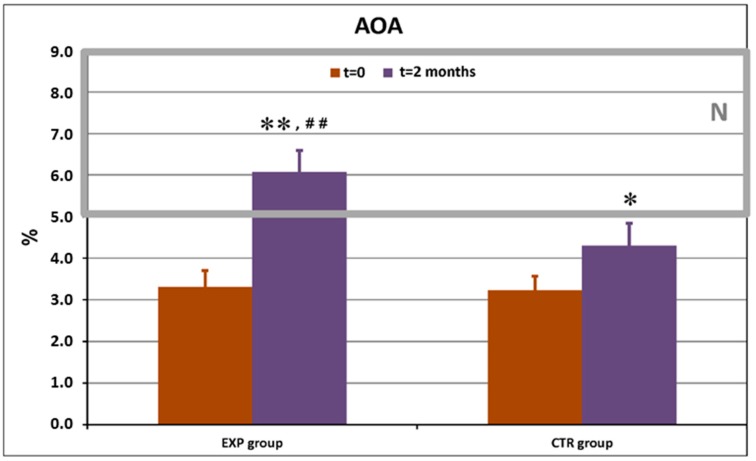
Total antioxidant activity (AOA, %) in gingival crevicular fluid in the experimental (EXP) and control (CTR) groups of patients before and after the trial. *N*: normal range of values obtained in healthy people. * *p* < 0.05 vs. baseline values; ** *p* < 0.01 vs. baseline values; ^##^ *p* < 0.01 vs. CTR.

Similar data were obtained with total AOA in GCF (Figure 4).

Total AOA values were initially lower-than-normal in both groups. The use of ETP led to normalization of this classical oxidative stress marker. CTP slightly increased the mean total AOA value, but it remained lower-than-normal (*p* < 0.05).

### 3.4. Differential Count of Periodontal Pathogens in GCF: In Vivo Effects of ETP and CTP

Quantitative real-time PCR analysis was carried out in selected patients from the experimental (n = 15) and control (n = 5) groups before entering the trial and after its succession. In total, concentrations of seven periodontal pathogens involved in pathogenesis of chronic inflammatory pathologies of periodontal tissues (gingivitis and periodontitis) were determined (Table 2). This data showed that both toothpastes eliminated several bacterial periodontal pathogens, diminishing their local gingival concentrations below a pathologically significant threshold. However, ETP was more efficient than CTP against three pathogens: *P.g.*, *F.n.*, and *P.i.*

### 3.5. The In Vitro Effects of CTP, ETP, and Its Active Herbal Ingredients on Bacterial Count and Bacterial Survival within Phagocytes (Intracellular Bacterial Killing)

To prove the hypothesis that the comparatively high clinical efficacy and the positive biochemical and molecular anti-inflammatory effects of ETP might be partly explained by its anti-bacterial properties, the experimental part of the research was designed using the in vitro systems to evaluate direct anti-bacterial action (bacteria plus toothpastes, bacteria plus herbal extracts) and indirect anti-bacterial effects in complex systems consisting of isolated human granulocytes and *S. aureus* strains treated or non-treated with toothpastes or herbal actives.

Among all substances studied to prove direct anti-bacterial effects towards *S. aureus*, both toothpastes exhibited direct bactericidal action, since its incubation with the bacteria substantially diminished the CFU (Table 2, the 2nd column).

Bacteria added to phagocytes, the *S. aureus* strain 1523, resistant to antibiotics with background catalase activity equal to 5.1 ± 0.1 units per 2 × 10^7^ cells, were selected.

The photo shows direct anti-bacterial effects of *M N1*: control tooth paste; *M N2*: experimental toothpaste; *A*: *Arnica montana*; *C*: *Chamomilla recutita*; *E,P*: *Echinacea purpurea*; *S*: *Salvia officinalis*.

Herbal actives at concentrations within ETP did not show any direct anti-bacterial action. At the same time, practically all active herbal ingredients and ETP strongly inhibited high catalase activity in the *S. aureus* strain 1382 resistant to antibiotics, while CTP only slightly inhibited bacterial catalase (Table 3, the 3rd column). Catalase activity in *S. aureus* directly correlated with bacterial survival after phagocytosis (correlation coefficient r = 0.9, *p* < 0.05) and inversely correlated with intracellular bacterial killing (Table 3, the 4th column).

The observed effect was proven on 10 *S. aureus* strains with different background activities of catalase. The catalase-containing bacteria pre-treated with ETP or with active herbal agents of ETP suppressed significantly bacterial catalase in all 10 *S. aureus* strains (Table 4) that correlated with much lower bacterial survival after phagocytosis (Table 5).

## 4. Discussion

Patients who entered this clinical laboratory study on the basis of inclusion and exclusion criteria were randomly assigned to experimental and control groups. The participants of the two groups were matched by sex, age, and the background clinical scores of gingivitis and early stage periodontitis (Table 1 and Figure 1). Clinical indices of gingivitis and periodontitis, such as PMA, Sillness–Loe, and CPITN indexes, statistically significantly improved in the experimental group of patients as compared to the beginning of the study and compared to the control group (Figure 1A,B,D). For the control group, the indices of gum health and plaque, such as plaque presence at the gingival border, number of teeth with thick plaque and tartar, inflammation, bleeding, and bleeding on the probe, showed a tendency of improvement; however, the clinical effects of CTP did not reach statistical significance. On these grounds, we come to the conclusion that ETP improved better than CTP clinical conditions of periodontal tissues diminishing gum inflammation (bleeding and redness), plaque presence, and initial symptoms of periodontitis.

Following these encouraging clinical data and observations done by doctors and patients, we proceeded to detailed laboratory examinations in order to elucidate the mechanisms underlying the remarkable clinical efficacy of the ETP.

Since poly-microbial infection is generally accepted as a major etiological factor of gingivitis and periodontitis [1,6,9], the primary lines of treatment include mechanical removal of microbial biofilms, as well as systemic and topical antibiotics. The need for repeated painful cleaning procedures and the acquired resistance of dental bacteria to antibiotics prompted an extensive search for alternative non-toxic, clinically- and cost-effective remedies, such as toothpastes, mouth rinses, and gingival gels, to prevent and decrease local bacterial overload. Recently, molecules inhibiting biofilm (plaque) formation have been suggested as having preventing and therapeutic potentials for gingivitis and periodontitis. Among the most effective disrupters of microbial biofilms, flavonoids, 2-aminoimidazole alkaloids, and halogenated furanones of plant origin have been identified [62]. Herbal constituents of ETP are well known for their numerous positive health effects. All four medicinal herbs included in the formulae of ETP have acceptable safety profiles to be used for topical application. They are also praised as exerting anti-bacterial, anti-inflammatory, and anti-oxidant effects in vitro and in vivo at low non-toxic concentrations [44,45,46,47,48,49,50,51,52,53,54,55,56,57] [and the Introduction herein]. First of all, direct anti-bacterial activity of ETP, CTP, and herbal extract-constituents of ETP was determined in bacterial cultures of *S. aureus* (Table 2, column 2 with Photo). It could be noted that ETP was very efficient in killing/blocking bacterial growth. ETP was approximately two orders of magnitude more efficient than CTP, while the herbal extracts exerted only moderate direct anti-bacterial effects. Our observations corresponded to published data on rather moderate anti-bacterial effects of both chemical constituents of CTP, disodium fluoride mono phosphate, and xylitol [30,31,63,64].

Bacterial levels higher than their individual threshold values greatly increase the risk of periodontal pathology. Thus, threshold concentration of *A.a.* is 10^4^ cells/mL, *P.g.*, *T.f.*, and *T.d.* is 10^5^ cells/mL, and *P.d.*, *F.n.*, and *P.i.* is 10^6^ cells/mL [16]. Our data on differential bacterial concentrations in GCF and plaque determined by the qrPCR method (Table 2) confirmed that both toothpastes under investigation decreased the load of bacteria bearing high risk of periodontal disease below their pathogenically relevant threshold. ETP was more effective against three (*P.g*., *F.n*., and *P.i*) out of seven bacteria investigated.

Although anti-bacterial action of individual herbs included into the ETP composition was rather modest, their combinations with other herbs and chemical anti-septics acquired synergy of action due to the fact that the herbs have different phytochemical compositions with different mechanisms of action towards bacteria. Moreover, herbal actives, such as secondary metabolites, could interact somehow with chemical anti-septics, thus invigorating their potential to fight against bacteria [19,45].

In the second set of the in vitro experiments, human granulocytes were added, and their ability to phagocyte and kill bacteria intracellularly afterwards was measured. Following the study design, *S. aureus* (antibiotic resistant strain with high initial catalase activity) was pre-incubated with the toothpastes and individual herbal constituents, before being subjected to phagocytosis by granulocytes. The results obtained show that CTP has a slight effect on bacterial catalase (11% suppression), as well as on intracellular survival of engulfed bacteria (Table 3, columns 3 and 4), while ETP and all herbal ingredients of ETP were extremely suppressive to bacterial catalase and intracellular bacterial survival. The degree of extract-induced stimulation of intracellular bacteria l killing and suppression of bacterial catalase was in order: *Salvia* > *Echinacea* > *Arnica* ≥ *Chamomile*. To avoid arguments that the results might reflect peculiarities of the *S. aureus* strain used, the experiment was repeated with the mixture of 10 different *S. aureus* strains different in their antibiotic resistance and background catalase activity (Table 4). The mixture of these strains had moderate average background catalase activity (2.38 ± 0.90 U/mL) (Table 5). For pre-incubation with ETP, with 3 out of 4 individual herbal ingredients and the mixture of 4 herbal extracts, initial catalase activity of the bacterial mixture was statistically significantly suppressed (*p* < 0.05). In accordance with our previous results herein (Table 3, 3rd column), *Salvia* and *Echinacea* were the most effective bacterial-catalase-suppressing herbs.

Catalase is a major enzyme that inactivates hydrogen peroxide, which is known as a low molecular weight anti-bacterial agent and regulator of a microbiota pattern [65,66]. Anti-bacterial hydrogen peroxide is mainly produced during the oxidative burst of phagocytes induced by bacteria [41,66]. Recently, a key role of granulocytes in pathogenesis of gingivitis and periodontitis has been recognised [2,3]. The generation of H_2_O_2_ by granulocytes (oxidative burst) is a key host defence against *Staphylococcus aureus*, *Escherichia coli*, *Porphyromonas gingivalis*, and other pathogenic bacteria [41,67,68,69,70]. The microbes become resistant to an oxidative burst owing to their innate and acquired capacity of adaptation to the host defence [67,71,72,73]. For example, to overcome oxidative stress with bactericidal action, the induction of microbial antioxidant enzymes, such as catalase and superoxide dismutase, occurs [67,70,74,75,76,77]. Molecular mechanisms of the adaptive response to oxidative stress in bacteria are complex and controlled by transcriptional factors: PerR, a sensor of hydrogen peroxide [70,71] and OxyR, a redox-dependent regulatory protein identified in *P. gingivalis*, in the majority of Gram-negative and several Gram-positive bacteria [72,73].

In the in vitro biofilm system, selective anti-bacterial activity of a combination of 1450 ppm NaF and stannous fluoride against *A. actinomycetemcomitans*, *P. gingivalis*, and *F. nucleatum* has been demonstrated [78]. However, both stannous fluoride and sodium monofluorophosphate were ineffective in the prevention of gingivitis [79] and exerted high tissue retention and toxicity [32,33]. Ethanol extracts of *Rosemarinus officinalis* L. [80], *Moringa oleifera* [81], and fermented syrup of *Carica papaya* [41] have shown remarkable anti-bacterial and anti-inflammatory potentials in the absence of any cytotoxic or genotoxic effects.

The herbal actives of ETP suppressed a microbial defence against an oxidative burst thus, bacteria were effectively killed intracellularly [41]. Striking a resemblance to the catalase-suppressing effects observed for ETP, plant extracts used in ETP and standardised fermented papaya gel [41] suggests that polyphenols, secondary metabolites in abundance in all these plant products, could be excellent anti-bacterials, acting at the level of granulocyte-bacteria interaction. Reactive oxygen and reactive nitrogen species (ROS and RNS, respectively) represent the first line of the anti-microbial host defence [19]. They are produced in great excess during primary oxidative responses of phagocytes to fight bacteria, viruses, parasites, etc. Local and generalized oxidative stress have, for a long time, been considered a molecular hallmark of gingivitis and periodontitis [18,23,25,26,41]. Here, we observed highly increased vs normal range values of background levels of nitrite and nitrates (RNS) in GCF of all recruited patients with clinically confirmed gingivitis and mild periodontitis (Figure 3). While the use of ETP resulted in complete normalisation of the nitrite/nitrate radio (*p* < 0.01 vs. day 0 and vs. control group), CTP induced a less evident decrease as compared to the beginning of the study (*p* < 0.05 vs. day 0). On the other hand, total antioxidant activity in GCF was lower-than-normal in both groups at day 0 (Figure 4) and reached normal values by the cessation of the study in the ETP group (*p* < 0.01 vs. day 0 and vs. CTP group). Total AOA slightly increased but remained below the lower edge of normality (*p* < 0.05 vs. day 0). We suggested that the normal balance of pro- and anti-oxidants in GCF achieved after a 2-month-long use of ETP could be attributed to herbal constituents known for their antioxidant properties [46,48,51,56].

While ROS and RNS are low molecular weight mediators of inflammation [19], pro-inflammatory and anti-inflammatory cytokines, proteins produced by immune cells in response to biotic and abiotic stresses are regulators of cell–cell interactions during inflammatory responses. Any alteration of cytokine production inevitably leads to inappropriate adaptive stress responses, with periodontal bacteria among them [3,4,7,8].

The present study showed remarkable anti-inflammatory action of ETP (Figure 4) when full normalisation of the inflammatory (IL-1β, IL-6, and IL-17A) and anti-inflammatory (IL-10) cytokine patterns in GCF were achieved. Evident suppression of inflammation in both groups could be partly explained by decreased bacterial overload provided by chemical anti-bacterials and herbal ingredients. Our data correspond to publications on clinical outcomes of essential oil-containing oral care products [82], which provided additional benefits towards gingival inflammation and plaque formation as compared to the placebo or control toothpastes and mouth rinses.

**Figure 5:**
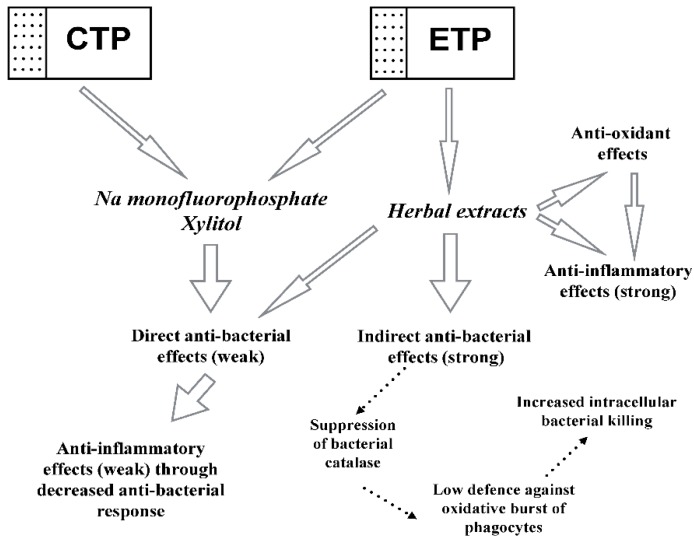
Mechanisms underlying clinical effects of experimental and control toothpastes. Control toothpaste (CTP); experimental toothpaste (ETP).

On the grounds of the results obtained, we conclude that the experimental toothpaste containing the following active ingredients (sodium mono-fluoride phosphate, xylitol, and medicinal Swiss herbs) was more clinically efficient than the control toothpaste, containing only sodium mono-fluoride phosphate and xylitol. We also assume that molecular events underlying greater-than-control clinical effects on patients suffering from gingivitis and initial stages of periodontitis could be additional indirect anti-bacterial action through suppression of bacterial defence against oxidative bursts of host phagocytes, as well as direct anti-inflammatory and anti-oxidant effects. Collectively, these events are graphically presented in Figure 5.

Both CTP and ETP contain sodium monofluorophosphate and Xylitol as active constituents with weak anti-bacterial action. Due to the diminished bacterial load induced by the two chemicals, the following inflammatory response from the host immune cells was decreased (weak anti-inflammatory effects). ETP additionally contains extracts of Swiss medicinal plants, which exert weak direct anti-bacterial and strong indirect anti-bacterial effects dependent on the inhibition of adaptive bacterial catalase activity in response to oxidative bursts in host phagocytes. Bacterial catalase inhibition leads to increased intracellular killing of bacteria engulfed during phagocytosis. Moreover, extracts of Swiss medicinal plants possess strong direct anti-oxidant and anti-inflammatory properties.

## 5. Conclusions

Although many efforts have been made to develop clinically and cost-effective protocols to prevent and treat gingivitis and early stages of periodontitis, none has proven to have sufficient efficacy, and the urgent need for safer treatments of high efficacy remains. As compared to classical chemical anti-septics traditionally used in oral care products, plant-derived extracts have better toxicity profiles towards human organisms. While anti-septic action of individual herbs included in experimental toothpaste is rather moderate, their combinations with other herbs and chemical anti-septics acquire synergy of action due to different bacterio-killing and bacterio-static mechanisms. Moreover, herbal actives, such as secondary metabolites, could interact with chemical anti-septics, thus invigorating their potential to fight against bacteria. Swiss medicinal plant-derived actives for oral care exert multiple positive effects, such as anti-inflammatory, anti-oxidant, direct anti-septic, and indirect anti-bacterial actions through the inhibition of bacterial defence against host phagocytes. On the grounds of the results obtained, it could be concluded that safe and efficient oral care products to prevent/treat gingivitis and early stage periodontitis should contain a combination of chemical and plant-derived anti-bacterials amid their different mechanisms of action.

## Figures and Tables

**Table 1:** Demographic distribution of patients with gingivitis and the initial stage of periodontitis (PD) in the treatment groups.

Group	Patients	Age, Years	Sex		Smokers	Diagnosis	
			M	F		Gingivitis	Initial PD
Experimental (conventional treatment + ETP twice a day for 60 days)	35	35–55	12	23	5	8	27
Control (conventional treatment + CTP twice a day for 60 days)	15	36–55	7	8	3	3	12

**Table 2:** Effects of ETP and CTP on the counts of high-risk and moderate-risk of periodontitis/gingivitis—inducing bacteria in the gingival crevicular fluid.

Bacteria, Risk Grade	Experimental Group (n = 15)		Control Group (n = 5)	
	Before (Cells/mL)	After 2 Months (Cells/mL)	Before (Cells/mL)	After 2 Months (Cells/mL)
*Aggregatibacter actinomycetem comitans, high risk*	7.9 × 10^5^ 2.2 × 10^6^ 1.3 × 10^7^ 1.9 × 10^5^ 1.3 × 10^5^ 1.2 × 10^5^	2.8 × 10^4^ 2.5 × 10^4^ 1.7 × 10^6^ 0 1.1 × 10^5^ 0	1.6 × 10^3^ 2.4 × 10^4^	0 6.5 × 10^4^
*Porphyromonas gingivalis, high risk*	2.8 × 10^6^ 1.2 × 10^6^ 1.9 × 10^5^ 1.2 × 10^6^ 2.7 × 10^7^	0 1.4 × 10^3^ 0 0 3.3 × 10^6^	2.9 × 10^6^ 2.0 × 10^4^ 1.6 × 10^5^ 8.5 × 10^3^	2.1 × 10^4^ 1.0 × 10^3^ 4.8 × 10^4^ 0
*Porphyromonas endodontalis, moderate risk*	7.8 × 10^3^ 1.2 × 10^4^ 1.2 × 10^4^ 8.4 × 10^5^ 6.9 × 10^5^ 1.2 × 10^4^ 1.2 × 10^4^ 1.4 × 10^4^ 1.8 × 10^4^ 3.7 × 10^6^	0 0 0 1.4 × 10^5^ 0 0 0 0 0 9.8 × 10^3^	1.2 × 10^4^ 1.2 × 10^4^	0 0
*Treponema denticola, high risk*	3.3 × 10^5^ 1.8 × 10^6^ 3.3 × 10^5^ 7.9 × 10^5^ 3.8 × 10^5^ 3.3 × 10^5^ 1.8 × 10^5^ 1.3 × 10^5^ 2.3 × 10^7^	0 3.7 × 10^3^ 0 0 1.1 × 10^3^ 0 0 1.1 × 10^2^ 2.7 × 10^6^	5.9 × 10^5^ 2.0 × 10^3^ 3.3 × 10^5^ 3.3 × 10^5^	1.9 × 10^5^ 0 0 0
*Tanerella forsythia, high risk*	1.2 × 10^6^ 2.6 × 10^5^ 3.2 × 10^5^ 3.7 × 10^4^ 5.8 × 10^5^ 4.8 × 10^6^ 5.4 × 10^3^ 6.0 × 10^5^ 7.5 × 10^4^ 7.5 × 10^4^ 3.2 × 10^7^	0 1.4 × 10^3^ 0 0 3.5 × 10^5^ 2.2 × 10^5^ 0 3.4 × 10^4^ 0 0 6.6 × 10^4^	1.9 × 10^6^ 1.3 × 10^6^ 4.6 × 10^4^	0 8.7 × 10^5^ 5.9 × 10^3^
*Prevotella intermedia, moderate risk*	5.8 × 10^5^ 4.5 × 10^5^ 5.8 × 10^5^ 4.0 × 10^3^ 1.2 × 10^5^ 1.9 × 10^7^	0 0 0 0 6.4 × 10^4^ 0	1.4 × 10^5^	4.5 × 10^6^
*Fusobacterium nucleatum, moderate risk*	6.6 × 10^5^ 1.0 × 10^3^ 1.4 × 10^5^ 1.2 × 10^5^ 1.4 × 10^5^ 1.6 × 10^5^ 4.2 × 10^4^ 6.4 × 10^5^ 8.3 × 10^6^ 6.1 × 10^5^ 2.1 × 10^5^ 3.6 × 10^5^ 6.6 × 10^6^ 4.6 × 10^6^ 6.2 × 10^5^	1.6 × 10^2^ 0 0 1.5 × 10^2^ 2.4 × 10^2^ 0 0 3.6 × 10^4^ 1.6 × 10^5^ 1.4 × 10^4^ 1.4 × 10^5^ 0 2.6 × 10^2^ 2.1 × 10^2^ 0	4.4 × 10^5^ 1.7 × 10^5^ 7.9 × 10^4^ 8.0 × 10^4^ 5.3 × 10^3^	0 3.5 × 10^4^ 5.9 × 10^3^ 3.2 × 10^5^ 5.0 × 10^3^

**Table 3:** Direct anti-bacterial effects of ETP, CTP, and herbal actives of ETP and their action towards microbial catalase activity and intracellular microbial survival.

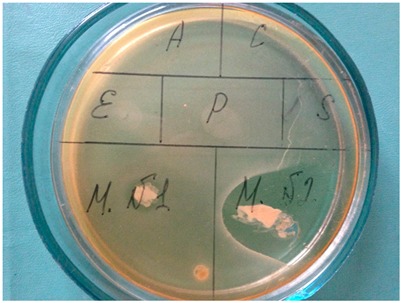			
Sample	Direct Anti-Bacterial Effect	Bacterial Catalase Activity after Pre-Treatment with Toothpaste/Herbal Extract (% of Inhibition)	Number of Bacteria Survived in Phagocytes after Pre-Treatment, Cells/mL
Negative control (no pre-treatment)	5 × 10^6^	5.1 (0%)	5 × 10^6^
Control toothpaste (CTP)	10^5^	4.5 (11.8%)	5 × 10^5^
Experimental toothpaste (ETP)	5 × 10^3^	3.1 (39.2%)	5 × 10^2^
Chamomile leaves extract	10^6^	2.7 (47.0%)	10^4^
Salvia leaves extract	10^6^	1.9 (63.7%)	10^2^
Arnica flower extract	10^6^	2.5 (50.9%)	10^3^
Echinacea flower extract	10^6^	2.8 (54.9%)	10^3^

**Table 4:** The sources and characteristics of *S. aureus* strains used in experiments on phagocytosis, intracellular killing, catalase-inhibiting, and anti-microbial activity of ETP and CTP.

Strain Number	Source of Isolation	Resistance to 5–10 Antibiotics	Catalase Activity (Units/2 × 10^7^ Bacteria)
1523	Throat, tonsils (chronic tonsillitis)	+++	**5.1**
1546	Oral epithelia (stomatitis)	−	**2.1**
1549	Throat, tonsils (chronic tonsillitis)	++	**3.7**
1555	Oral epithelia (stomatitis)	+	**2.3**
1561	Nasal sinuses (sinusitis)	−	**2.2**
1612	Oral epithelia (stomatitis)	−	**2.2**
1620	Throat, tonsils (chronic tonsillitis)	+	**2.6**
1643	Throat, tonsils (chronic tonsillitis)	−	**2.2**
1670	Oral epithelia (stomatitis)	−	**2.1**
1780	Throat, tonsils (chronic tonsillitis)	−	**2.0**

**Table 5:** The in vitro effects of ETP, CTP, and active ingredients of ETP to catalase activity in *S. aureus* strains.

Sample	Initial Catalase Activity (U/mL) in *S. aureus* Strains (Mean ± S.D.)	Catalase Activity (U/mL) in *S. aureus* Strains after Pre-Treatment with Toothpastes or Active Herbal Ingredients
Control toothpaste (CTP)	2.38 ± 0.90	2.27 ± 0.93
Experimental toothpaste (ETP)	2.38 ± 0.90	1.52 ± 0.13 *
Chamomile leaves extract	2.38 ± 0.90	1.81 ± 0.21
Salvia leaves extract	2.38 ± 0.90	1.28 ± 0.71 *
Arnica flower extract	2.38 ± 0.90	1.68 ± 0.70
Echinacea flower extract	2.38 ± 0.90	1.27 ± 0.71 *
Mixture of extracts	2.38 ± 0.90	1.51 ± 0.42 *

* *p* < 0.05.
